# Parkinson’s Disease Diagnosis Using Laplacian Score, Gaussian Process Regression and Self-Organizing Maps

**DOI:** 10.3390/brainsci13040543

**Published:** 2023-03-24

**Authors:** Mehrbakhsh Nilashi, Rabab Ali Abumalloh, Sultan Alyami, Abdullah Alghamdi, Mesfer Alrizq

**Affiliations:** 1 UCSI Graduate Business School, UCSI University, No. 1 Jalan Menara Gading, UCSI Heights, Cheras, Kuala Lumpur 56000, Malaysia; 2 Centre for Global Sustainability Studies (CGSS), Universiti Sains Malaysia—USM, Penang 11800, Malaysia; 3 Department of Computer Science and Engineering, Qatar University, Doha 2713, Qatar; 4 Computer Science Department, College of Computer Science and Information Systems, Najran University, Najran 55461, Saudi Arabia; 5 Information Systems Department, College of Computer Science and Information Systems, Najran University, Najran 55461, Saudi Arabia

**Keywords:** Parkinson’s disease, UPDRS prediction, Laplacian score, self-organizing maps, Gaussian process regression

## Abstract

Parkinson’s disease (PD) is a complex degenerative brain disease that affects nerve cells in the brain responsible for body movement. Machine learning is widely used to track the progression of PD in its early stages by predicting unified Parkinson’s disease rating scale (UPDRS) scores. In this paper, we aim to develop a new method for PD diagnosis with the aid of supervised and unsupervised learning techniques. Our method is developed using the Laplacian score, Gaussian process regression (GPR) and self-organizing maps (SOM). SOM is used to segment the data to handle large PD datasets. The models are then constructed using GPR for the prediction of the UPDRS scores. To select the important features in the PD dataset, we use the Laplacian score in the method. We evaluate the developed approach on a PD dataset including a set of speech signals. The method was evaluated through root-mean-square error (RMSE) and adjusted R-squared (adjusted R²). Our findings reveal that the proposed method is efficient in the prediction of UPDRS scores through a set of speech signals (dysphonia measures). The method evaluation showed that SOM combined with the Laplacian score and Gaussian process regression with the exponential kernel provides the best results for R-squared (Motor-UPDRS = 0.9489; Total-UPDRS = 0.9516) and RMSE (Motor-UPDRS = 0.5144; Total-UPDRS = 0.5105) in predicting UPDRS compared with the other kernels in Gaussian process regression.

## 1. Introduction

Parkinson’s disease (PD) is a complex degenerative brain disease with increasing motor symptoms that can significantly impair patients’ quality of life [1,2]. Aging has been linked to a number of negative health consequences, including those affecting the nervous system [3]. The number of people affected by these conditions is expected to increase as the global population ages. The most significant risk factor for developing PD appears to be age. The disease is typically diagnosed in people over the age of 60 [4,5,6], but it can affect younger people as well; 20% of patients are diagnosed with PD before the age of 50. PD affects 6.3 million people worldwide [7], and the disease’s impact on quality of life and life expectancy, as well as social and monetary costs, are expected to grow as the population ages. According to the statistic, there will be 8.7 million PD patients by 2030 [8]. Furthermore, the statistic shows that the number of PD patients in the US is predicted to increase to around 1.8 million by 2030 [9]. There is no one specific test that can diagnose PD. Instead, a neurologist will examine a patient’s symptoms and medical history and perform a neurological examination in order to make a diagnosis.

Because of the high heterogeneity of PD, each individual may experience a variety of symptoms. Since the initial symptoms are mild, they can go undetected for long periods. Furthermore, at the diagnostic level, 60% of PD patients have a clear asymmetry of symptoms. There are numerous reported PD symptoms, both motor and nonmotor [10,11]. Constipation, sleep disorders, rapid eye movement (REM) sleep behavior disorders, bladder disorders (urinary incontinence) and anxiety are some examples of nonmotor symptoms. Note that non-motor symptoms can sometimes precede motor symptoms, and are thought to represent the disease’s early stage. The secondary symptoms can be freezing of gait, gait dysfunction, hallucination, smell dysfunction, thinking difficulties, dementia, sexual dysfunction and depression. Although there is no cure for PD, there are treatments available to help manage its symptoms. The goal of treatment is to either replace the dopamine that is missing in the brain or to correct the problems that are caused by the lack of dopamine. Patients may be unaware of this disorder’s most common symptom, which is reduced vocal loudness. In addition, people with Parkinson’s disease commonly suffer from dysphonia [12], which is vocal impairment and characterized by a breathy voice and harshness. As the disease progresses, patients may experience greater difficulty speaking.

The UPDRS, or unified Parkinson’s disease rating scale, which measures the severity and presence of symptoms of PD, is the most popular tool used by clinicians to measure PD symptom severity (but does not measure their underlying causes) [3,13,14]. The UPDRS scale consists of three sections that assess motor symptoms, activities of daily life and mentation, behavior and mood. Monitoring the progression of PD is essential for better patient-directed care [3,13,15,16]. A convincing method for accurately and effectively tracking the progression of PD at more frequent intervals with less expense and resource waste is remote monitoring. A growing option in general medical care is noninvasive telemonitoring, which may allow for reliable, affordable PD screening while potentially reducing the need for frequent clinic visits. As a result, the clinical evaluation of the subject’s condition is evaluated more accurately and the burden on national healthcare systems is reduced.

Machine learning has demonstrated to be effective in disease diagnosis [15,16,17,18,19,20,21,22,23,24,25,26,27]. There have been many methods for PD diagnosis; some of them are presented in Table 1. The findings for the methods presented in this table show that there is no research on the use of clustering, feature selection and prediction machine learning for the prediction of UPDRS. As seen from this table, the previous research was mainly developed using prediction learning techniques. The use of clustering techniques can be effective in developing a robust learning method for UPDRS prediction. Clustering is effective because it allows the PD diagnosis methods to identify groups of similar objects or data points in the PD dataset. By grouping similar data points, the underlying patterns and structures within the data can be better understood to make more informed decisions. Accordingly, this study aims to develop a new method using clustering, feature selection and prediction machine learning to predict UPDRS scores (Total-UPDRS and Motor-UPDRS) and simulate the relationship between the characteristics of speech signals (dysphonia measures) and UPDRS scores. In this research, Motor-UPDRS is the motor section of the UPDRS. In addition, Total-UPDRS is the full range of UPDRS as described in [13]. Our method is developed using the Laplacian score, Gaussian process regression (GPR), and self-organizing maps (SOM) techniques. The SOM technique is used to segment the data to handle large PD datasets. The models are then constructed using the GPR technique for UPDRS prediction. To select the important features in the PD dataset, we use the Laplacian score in the method. We perform several experiments on a PD dataset in the UCI machine learning archive, including a set of speech signals (dysphonia measures), to evaluate the developed method.

The remainder of this paper is organized as follows. In Section 2, the techniques incorporated in the proposed method are presented. Data analysis and method evaluations are performed in Section 3. In Section 4, the discussion section is presented. Finally, this work is concluded in Section 5.

## 2. Method

**Figure 1:**
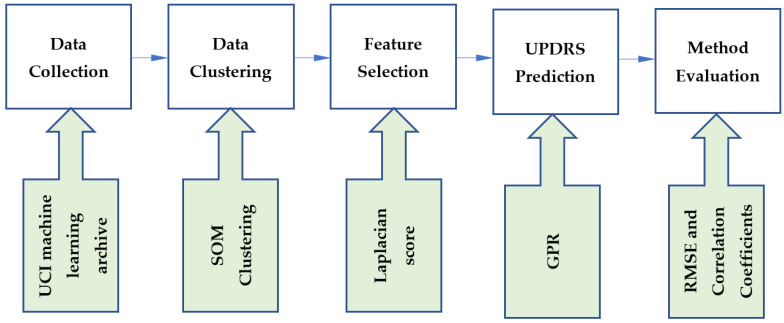
The steps of the proposed method.

This study developed a hybrid method using unsupervised, feature selection and supervised learning techniques. The steps of the proposed method are shown in Figure 1. The data were collected from the UCI machine learning archive. In the first step of our methodology, data were clustered using the SOM clustering technique. We then used the Laplacian score for feature selection. To perform UPDRS prediction, GPR was implemented on the generated clusters. The proposed method was evaluated using root-mean-square error (RMSE) and correlation coefficients. In this section, the techniques incorporated in the proposed method are introduced.

### 2.1. Gaussian Process Regression

The Gaussian process regression is a stochastic process that can be interpreted as probability distributions over functions with a number of random variables [80,81]. A joint Gaussian distribution exists for any finite range of these random variables. The Gaussian process regression is a machine learning approach that can be employed to deal with complex problems (e.g., nonlinear problems) [82]. It is developed on the basis of statistical theory and Bayesian theory [83]. This technique is widely used for regression problems [83,84].

A training dataset is required to establish a relationship between the input and output variables of the dataset. Assume that there is a dataset D with d-dimensional input vector $x_{i}\in R^{d}$ (d≥1) and with $y_{i}$ as the corresponding output. Then, we have:

(1) $$D=\left\{D_{i};\;i=1,\;2,\ldots ,\;p\right\}=\left\{\left(x_{i},y_{i}\right);\;i=1,\;2,\;\ldots ,p\right\}$$

Thus, the output vector $y={\left\{y_{i}\right\}}_{i=1:p}$ and matrix $X={\left\{x_{i}\right\}}_{i=1:p}$ are organized, respectively, for $y_{i}$ values and $x_{i}$ vector. Gaussian process regression employs a Gaussian prior which is parameterized through a covariance function $k\left(x,\;x^{\prime }\right)$ and the mean function m(x) to model a time series.

(2) $$y=f\left(x\right)∼GP\left(m\left(x\right),\;k\left(x,\;x^{\prime }\right)\right)$$

where m(x) is typically taken to be zero without affecting generality, and f(x) is known as the latent variable in the Gaussian process regression model.

The similarity between input data points, which is a crucial component of the Gaussian process regression model, is described by the covariance function $k\left(x,\;x^{f}\right)$. Different kernel functions are used in Gaussian process regression. A common kernel function used in Gaussian process regression is squared exponential (SE), which is represented as:

(3) $$k_{SE}\left(x,\;x^{\prime }\right)=\psi_{1}^{2}\exp\left(-\frac{{\left(x-x^{\prime }\right)}^{2}}{2\psi_{2}^{2}}\right)$$

where $\psi_{1}$ and $\psi_{2}$ indicate two hyperparameters that govern the accuracy of the output prediction. They need to be optimized in Gaussian process regression.

During the training phase of Gaussian process regression, the log-likelihood function in the following equation is maximized for the estimation of the kernel matrix’s parameters K:

(4) $$\psi_{opt}=arg\;max_{\psi }\{log\;p(y|X,\;\psi )\}=arg\;\;max\;\{-\frac{1}{2}\;log\;\left|K+Reject_{p}^{2}I\right|-\frac{1}{2}{\left(y-m\right)}^{T}{\left(K+\sigma_{p}^{2}I\right)}^{-1}\left(y-m\right)-\frac{p}{2}\;\log(2\pi )$$

where in the above equation, $\sigma_{p}^{2}$ indicates the variance of the noise. In this function, p indicates the number of test data points.

Because the log-likelihood function is convex, the gradient descent algorithm can solve Equation (4).

After training the model, at test points $X_{*}$, the posterior distribution of $f_{*}=f\left(X_{*}\right)$ will be obtained as:

(5) $$\begin{array}{ll} f_{*}|X,y,X_{*}∼ & N\left(\bar{f},\;cov\left(f_{*}\right)\right)\\ & \bar{f}=K\left(X_{*},\;X\right){\left(K\left(X,\;X\right)+\sigma_{n}^{2}I\right)}^{-1}\left(y-m\left(X\right)\right)cov\left(\bar{f}\right)+m\left(X_{*}\right)=K\left(X_{*},\;X_{*}\right)-K\left(X_{*},\;X\right){\left(K\left(X,\;X\right)+\sigma_{n}^{2}I\right)}^{-1}K\left(X,\;X_{*}\right) \end{array}$$

In the above formula, cov $\left(\bar{f}\right)$ indicates the prediction variance and $\bar{f}$ denotes the prediction mean. 

### 2.2. SOM

As an unsupervised learning algorithm, SOM [85] is used to cluster and visualize high-dimensional datasets simultaneously [86,87,88,89,90,91]. The self-organization process used by the learning algorithm was biologically inspired by the cortex brain cells. In contrast to the error-correction learning used in feedforward neural networks, this type of learning is referred to as competitive learning. A map is a grid-organized neural network made up of interconnected nodes, also known as cells, neurons or units. For visual purposes, the grid topology is typically two-dimensional [92,93], but it can have any topology. A prototype vector from the high-dimensional input space where the data live is assigned to each cell. The prototypes are updated to fit the training set during an iterative learning process; when a prototype is updated, the prototypes associated with neighboring cells are also updated using a specific weight. As the distance between grid cells increases, the weights decrease and the cells on the map near each other are linked to prototype vectors in the input space near each other. This allows the map to preserve the topology of the space. The resulting map, after convergence, allows for efficient visualization of the high-dimensional input space on a low-dimensional map. Because of its ease of use and interpretable results, SOM is a popular clustering and visualization tool. The SOM procedure for clustering is presented in Algorithm 1. In this algorithm, input patterns $X=\left\{\vec{x_{1}}\;,\ldots ,\;\vec{x_{N}}\right\}$ are considered for the data clustering in SOM. Number of iterations $t_{\;\max\;}$, learning rate ε(t) and neighborhood function σ(t) must be initialized in SOM to perform the data clustering. Note that each neuron $w_{i}$ represents an arbitrary number of input patterns. The output of SOM is a trained map and clustered input patterns. In Algorithm 1, the learning rate and radius of the neighborhood must both decrease at a constant rate for the algorithm to converge.
**Algorithm 1: SOM Procedure****Inputs:**$\;\mathrm{Input}\;\mathrm{patterns}\;X=\left\{\vec{x_{1}}\;,\ldots ,\;\vec{x_{N}}\right\}$, number of iterations $t_{\max}$, learning rate ε(t), neighborhood function σ(t) **Output:** Trained map and clustered input patterns Randomly initialize neurons, $w_{i}\in {\mathbb{R}}^{D},$
∀i **For** $t=1\;\mathrm{to}\;t_{\max}$
**Do**  ■An input pattern is randomly drawn, $\vec{x_{d}}$→$p={\;\arg\;\min}_{i}\left\{\left\|\vec{x_{d}}-\vec{w_{i}}\right\|\right\}$ (the neuron closer to the input pattern is selected)→$\vec{w_{i}}=\vec{w_{i}}+\varepsilon \left(t\right)\cdot h_{ip}\left(t\right)\cdot \left(\vec{x_{d}}-\vec{w_{i}}\right),\forall i$ (the winning neuron p is updated), $h_{ip}\left(t\right)$ indicates the neighborhood influence function where $h_{ip}\left(t\right)=exp-\frac{{\left|{\vec{a}}_{i}-{\vec{a}}_{p}\right|}^{2}}{\;\sigma^{2}\left(t\right)}$ (for two lattice vectors ${\vec{a}}_{i}$ and ${\vec{a}}_{p}$)→$\sigma \left(t\right)=\sigma_{0}{\left(\sigma_{f}/\sigma_{0}\right)}^{t/t_{\;\max\;}}$(the size of the radius is updated)→$\varepsilon \left(t\right)=\varepsilon_{0}{\left(\varepsilon_{f}/\varepsilon_{0}\right)}^{t/t_{\;\max\;}}$(the learning rate is updated)→t←t+1 (the number of iterations is incremented)

### 2.3. Laplacian Score for Feature Selection

The Laplacian score is based on Laplacian eigenmaps [94] and is considered a graph-based feature selection method. The Laplacian score models the data’s local geometrical structure [95,96] with a k-nearest neighbor (k-NN) graph. Consider a dataset $X=x_{1},\ldots ,x_{N}$; to approximate the dataset’s manifold structure, a k-NN graph is constructed, which contains an edge with weight $W_{ij}$ between $x_{i}$ and $x_{j}$ if $x_{i}$ is one of $x_{j}$’s k-nearest neighbors, or conversely. There are several similarity-based methods for determining edge weights. The Euclidean distance is one of the popular similarity metrics to measure the distance between two vectors [97,98]. Thus, for $x_{i}$ and $x_{j}$ and with τ as a suitable constant, we can define the weight matrix W as follows:

(6) $$W_{ij}=\{\begin{array}{l} e^{-\frac{\|x_{i}-x_{j}{\|}^{2}}{\tau }},\;\mathrm{if}\;x_{i}\;\mathrm{and}\;x_{j}\;\mathrm{are}\;\mathrm{neighbors}\\ 0,\;\mathrm{otherwise}. \end{array}$$

Two data points can only be considered close to one another on a feature if and only if there is an edge connecting them. To select a good feature, the following objective function needs to be minimized:

(7) $$SC_{Ls}=\frac{\sum_{ij}^{\;}{\left(f_{ri}-f_{rj}\right)}^{2}W_{ij}}{Var\left(f_{r}\right)}$$

where $f_{ri}$ indicates the *i*th sample of the *r*th feature in the dataset and $f_{r}=$ ${\left(f_{r1},\;\ldots \;,\;f_{rN}\right)}^{T}$, $Var\left(f_{r}\right)$ denotes the estimated variance of $f_{r}$. In order to maximize representative power, larger variance features are preferred.

Accordingly, we can obtain the variance of weight using the following equation.

(8) $$Var\left(f_{r}\right)={\tilde{f}}_{r}^{T}D{\tilde{f}}_{r}\text{’}\tilde{f}=f_{r}-\frac{f_{r}^{T}D1}{1^{T}D1}1\;\mathrm{where}\;D_{ii}=\sum_{j}^{\;}W_{ij}$$

where $D_{ii}$ is a diagonal matrix, and the corresponding degree matrix of W and 1 is a nonzero constant vector.

Accordingly, the mean of each feature $f_{r}$ by Equation (8) is removed. This is carried out to avoid assigning a zero Laplacian score to a nonzero constant vector, such as 1, because such a feature obviously contains no information. For a good feature, we have:

(9) $$\overset{\;}{\underset{ij}{\sum }}{\left(f_{ri}-f_{rj}\right)}^{2}W_{ij}=2f_{r}^{T}Lf_{r}=2{\tilde{f}}_{r}^{T}L{\tilde{f}}_{r}$$

where a bigger $W_{ij}$ indicates a smaller $\left(f_{ri}-f_{rj}\right)$, L is the Laplacian matrix and L=D−W.

Accordingly, the *r*th feature’s Laplacian score is reduced to

(10) $$SC_{Ls}=\frac{{\tilde{f}}_{r}^{T}L{\tilde{f}}_{r}}{{\tilde{f}}_{r}^{T}D{\tilde{f}}_{r}}$$

## 3. Data Analysis and Method Evaluation

In this research, we used Parkinson’s telemonitoring dataset [13] to evaluate the proposed method. Table 2 presents the features of this dataset. The dataset was published online in 2009 at the UCI machine learning archive. This dataset consists of around 200 recordings per patient from 42 people (28 men and 14 women) with early-stage PD, which makes a total of 5875 voice recordings. Each patient’s phonations of the sustained vowel /a/ are recorded. Parkinson’s telemonitoring dataset includes two outputs Motor-UPDRS and Total-UPDRS and sixteen biomedical voice measures (F1–F16). They are presented in Table 2. A full description of these features is presented in [13].

The dataset was clustered by SOM for different topology maps. The SOM clustering quality was assessed by the Silhouette index. To measure the separation between the resulting clusters, this index can be used. The silhouette index of the object $x_{i}$ is defined as:

(11) $$\mathrm{SI}\left(x_{i}\right)=\frac{b\left(x_{i}\right)-a\left(x_{i}\right)}{max\;\left(b\left(x_{i}\right),a\left(x_{i}\right)\right)}$$

where $a\left(x_{i}\right)$ denotes the distance of $x_{i}$ to its own cluster, which is characterized as the average distance between the object $x_{i}$ and all the other objects in its own cluster h as:

(12) $$a\left(x_{i}\right)=\frac{\sum_{j=1}^{n}w_{jh}d_{E}\left(x_{i},\;x_{j}\right)}{n_{h}-1}$$

where $n_{h}$ denotes the number of data points in the cluster $h,\;d_{E}\left(i,j\right)$ denote the squared Euclidean distance, $w_{jh}$ indicates the indicator function ($w_{jh}=1,x_{i}$ is in $c_{h}$; $w_{jh}=0,\;x_{i}$ is not in $c_{h})$.

The minimum average distance between the object $x_{i}$ and every other object in a cluster, excluding the cluster to which the object $x_{i}$ belongs, is defined by b(i). b(i) is calculated by:

(13) $$b\left(x_{i}\right)=\min\left(\frac{\sum_{j=1}^{n}w_{jl}d_{E}\left(x_{i},x_{j}\right)}{n_{l}}\right)$$

Accordingly, SI $\left(x_{i}\right)\in \left[-1,1\right]$. When SI $\left(x_{i}\right)$ is close to 1, the element $x_{i}$ is assigned to the correct cluster. When this value is close to −1, the object $x_{i}$ is in the incorrect cluster because the neighboring cluster is a better option than the selected cluster. The validity of the entire clustering can then be evaluated using the silhouette index, which is defined as:

(14) $$\bar{\mathrm{SI}}=\frac{1}{n}\overset{\;}{\underset{i\in X}{\sum }}SI\left(x_{i}\right)$$

**Figure 2:**
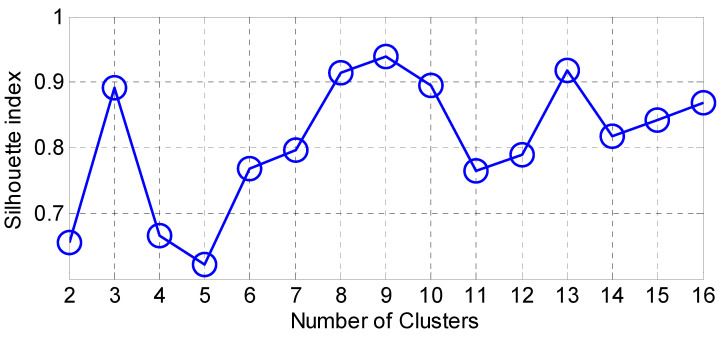
The results for silhouette index.

**Figure 3:**
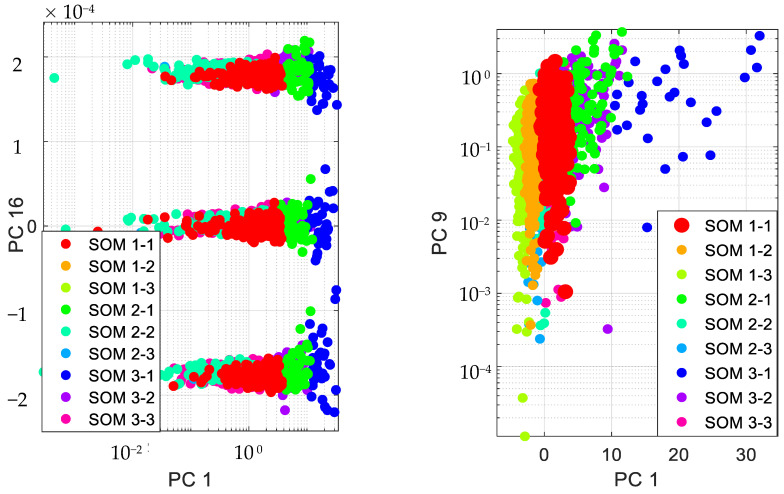

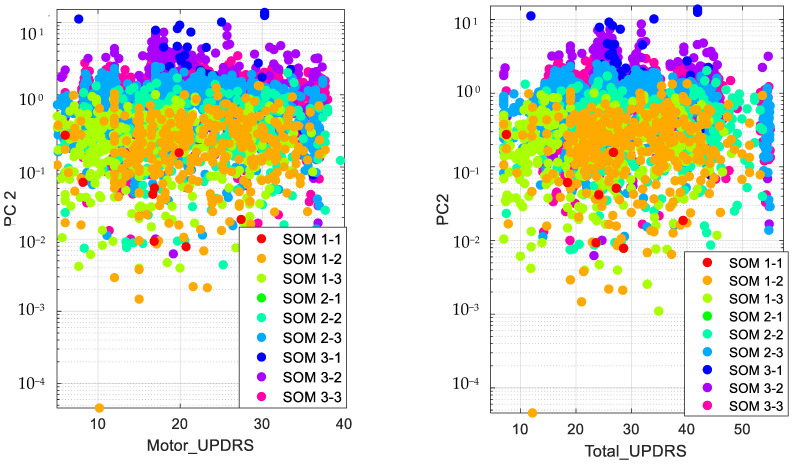
Visualizing SOM clusters.

Accordingly, we present the results for the silhouette index in Figure 2. As seen from this figure, nine clusters in SOM provide the best silhouette index, as the highest value for $\bar{\mathrm{SI}}$ is obtained for nine clusters. Hence, we clustered the PD data in nine clusters, as presented in Figure 3. The clusters in this figure are visualized using different PCs (principal components), which are PC 1, PC 9 and PC 16. In addition, in this figure, Total-UPDRS and Motor-UPDRS are visualized using PC 2 in nine clusters of SOM. We also provide the cluster centroids in Table 3. In this table, nine clusters are presented along with the centroid for each feature of the PD dataset.

**Figure 4:**
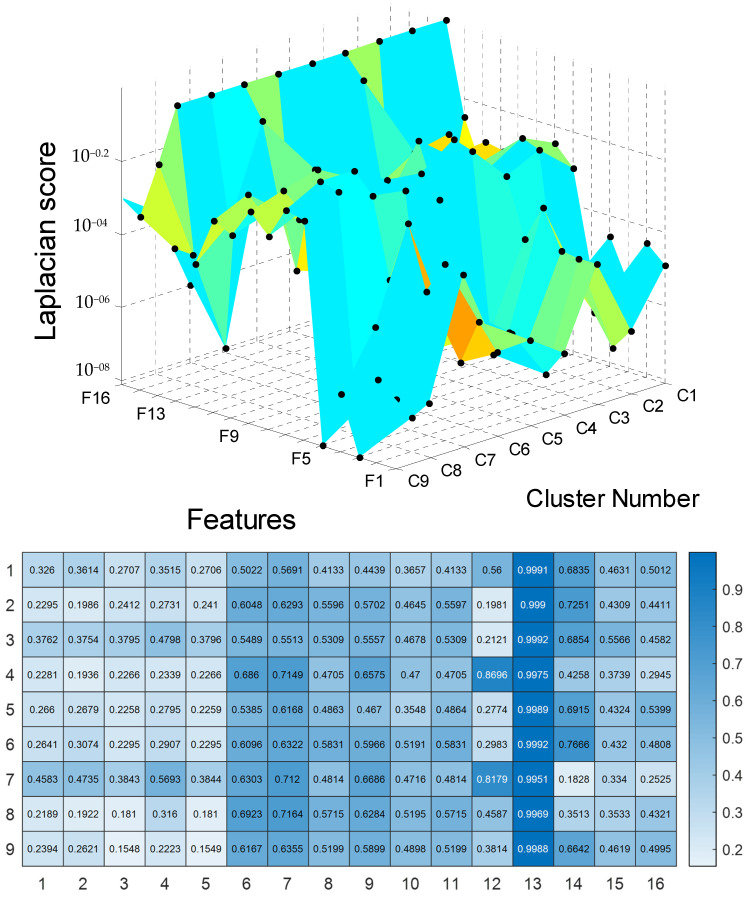
The results for Laplacian score.

To perform UPDRS prediction in each cluster of SOM, we first used the Laplacian score technique for feature selection. The results of the feature selection are presented in Figure 4 and Table A1 in Appendix A. For these results, the features are ranked for unsupervised learning using Laplacian scores. According to the results, a large score value indicates that the corresponding PD feature is important.

The selected features of nine clusters of Parkinson’s telemonitoring dataset were used in Gaussian process regression for UPDRS score (Total-UPDRS and Motor-UPDRS) predictions. In this study, the 10 most important features were selected for UPDRS prediction in each cluster. As a result, there were nine clusters, each of which included ten important features for UPDRS prediction.

The method was run on Microsoft Windows 10 Pro and a laptop with Processor Intel(R) Core(TM) i7-6700HQ CPU @ 2.60GHz, 2592 Mhz, four core(s) and eight logical processor(s). A 5-fold cross-validation approach in the hyperparameter optimization to avoid overfitting was used in training the models in Gaussian process regression. For example, to combine RMSE and five-fold cross-validation, we applied the following steps:

- Dividing the data into five equal parts;
- Training the model on four parts of the data and testing it on the fifth part, calculating the RMSE for that fold;
- Repeating step 2 for all five folds;
- Calculating the average RMSE across all five folds. This provided an estimate of the model’s overall performance.

The nine models were assessed using RMSE and correlation coefficients. The highest value of adjusted R-squared (adjusted R² or the coefficient of determination) means perfection. Lower values of RMSE reflect better performance by the predictor. The RMSE is presented in Equation (15).

(15) $$RMSE=\sqrt{\overset{N}{\underset{n=1}{\sum }}\frac{1}{N}{\left[\hat{UPDRS}\left(n\right)-UPDRS\left(n\right)\right]}^{2}}$$

This metric is defined for a testing vector of length N, actual UPDRS and forecasted $\hat{UPDRS}$. In this study, four kernels were used in Gaussian process regression: rational quadratic kernel, squared-exponential kernel, exponential kernel and Matérn 5/2 kernel. In Table 4 and Table 5, we present the average results of R-squared and RMSE for Total-UPDRS and Motor-UPDRS, respectively, in Parkinson’s disease. The results are provided for maximum, minimum and mean values of R-squared and RMSE. The results presented in Table 4 and Table 5 clearly show that SOM + Laplacian score + Gaussian process regression (exponential kernel) provide the best results for R-squared and RMSE in predicting Total-UPDRS and Motor-UPDRS compared with the SOM + Laplacian score + Gaussian process regression (squared-exponential kernel), SOM + Laplacian score + Gaussian process regression (rational quadratic kernel) and SOM + Laplacian score + Gaussian process regression (Matérn 5/2 kernel). Furthermore, the findings reveal that SOM + Laplacian score + Gaussian process regression (Matérn 5/2 kernel) provided the largest prediction errors for Total-UPDRS and Motor-UPDRS.

## 4. Discussion

Machine learning has significant implications for PD. Researchers and healthcare providers can gain deeper insights into the disease by leveraging machine learning algorithms, allowing for earlier diagnosis, personalized treatment plans and improved symptom management. Early detection of PD is critical because early intervention can help slow disease progression and improve patient outcomes. By analyzing patient data and identifying specific patterns associated with the disease, machine learning can aid in the early detection of PD. Machine learning can also be used to create personalized treatment plans for patients with PD, taking into account individual patient data such as medical history, genetic information and response to previous treatments. This can assist healthcare providers in tailoring treatments to each patient’s specific needs, improving treatment outcomes and quality of life. Furthermore, machine learning can help manage PD’s symptoms, particularly through remote monitoring. Wearable devices with machine learning algorithms can monitor changes in motor symptoms and alert healthcare providers if necessary, enabling more proactive and responsive care. Overall, the implications of machine learning for PD are promising, opening up new avenues for disease diagnosis, treatment and management that can improve patient outcomes and quality of life.

## 5. Conclusions

The use of voice measurements has been an effective way for remote tracking of UPDRS. It eases the clinical monitoring of patients and increases the chances of early diagnosis of PD. Machine learning has been widely used in the analysis of speech signals in the diagnosis of PD. Accordingly, there have been many attempts in improving the accuracy of machine learning methods in this context. This study relied on feature selection, clustering and prediction learning techniques in improving the accuracy of PD diagnosis systems. We used the Laplacian score technique as a feature selection technique, SOM as a clustering technique based on the neural network approach, and Gaussian process regression as a prediction learning technique in the development of a new method for UPDRS prediction. SOM discovered nine clusters from the PD dataset. In each cluster of SOM, the most important features were selected by the Laplacian score technique for UPDRS precision by Gaussian process regression. Gaussian process regression was applied using different kernels, namely the rational quadratic kernel, squared-exponential kernel, exponential kernel, and Matérn 5/2 kernel. The method was evaluated through RMSE and adjusted R-squared. The results revealed that SOM + Laplacian score + Gaussian process regression (exponential kernel) provide the best results for R-squared and RMSE in predicting Total-UPDRS and Motor-UPDRS compared with the SOM + Laplacian score + Gaussian process regression (squared-exponential kernel), SOM + Laplacian score + Gaussian process regression (rational quadratic kernel) and SOM + Laplacian score + Gaussian process regression (Matérn 5/2 kernel). Although the proposed method has accurately predicted the UPDRS through a set of selected features by Laplacian score, this method can be further improved by optimizable Gaussian process regression. In addition, the use of incremental Gaussian process regression is greatly suggested in the development of the proposed method for online learning of PD data. The incremental use of Gaussian process regression will significantly improve the efficiency of the proposed method, particularly when there are big datasets for PD with many features of speech signals.

## Figures and Tables

**Table 1:** Related works on PD diagnosis.

Method	References
K-nearest neighbor	[28,29,30,31,32,33,34,35]
Neural networks	[32,36,37,38,39,40,41,42,43,44,45,46,47,48,49,50,51]
ANFIS	[16,52,53,54]
Fuzzy logic	[28,30,55,56,57]
Genetic programming	[46,58,59,60,61]
Clustering	[62,63,64,65]
Principal component analysis	[66,67,68,69,70]
Deep learning	[15,31,51,71,72,73,74,75,76,77,78,79]

**Table 2:** The Total- and Motor-UPDRS, and 16 biomedical voice measures in Parkinson’s telemonitoring dataset.

Feature ID	Feature Name	Mean	Min	Max	SD
F1	HNR	21.679	1.659	37.875	4.291
F2	Jitter: DDP	0.009	10 × 10^4^	0.173	0.009
F3	RPDE	0.541	0.151	0.966	0.101
F4	MDVP: Jitter (%)	0.006	8 × 10^4^	0.1	0.006
F5	MDVP: Shimmer (dB)	0.311	0.026	2.107	0.230
F6	PPE	0.220	0.022	0.732	0.092
F7	MDVP: Shimmer	0.034	0.003	0.269	0.026
F8	MDVP: Jitter (Abs)	4 × 10^5^	2 × 10^6^	4 × 10^4^	3 × 10^5^
F9	DFA	0.653	0.514	0.866	0.071
F10	Shimmer: APQ3	0.017	0.002	0.163	0.013
F11	MDVP: Jitter:PPQ5	0.003	4 × 10^4^	0.069	0.004
F12	Shimmer: APQ11	0.028	0.003	0.276	0.020
F13	NHR	0.032	3 × 10^4^	0.749	0.060
F14	MDVP: Jitter:RAP	0.003	3 × 10^4^	0.057	0.003
F15	Shimmer: DDA	0.052	0.005	0.488	0.040
F16	Shimmer: APQ5	0.020	0.002	0.167	0.017
-	Total-UPDRS [after-6-months]	29.57	7	54	11.92
-	Motor-UPDRS [after-6-months]	29.57	5	41	9.17
-	Total-UPDRS [after-3-months]	29.36	7	55	11.82
-	Motor-UPDRS [after-3-months]	21.69	6	38	9.18
-	Total-UPDRS [baseline]	26.39	8	54	10.8
-	Motor-UPDRS [baseline]	19.42	6	36	8.12

**Table 3:** Cluster centroids.

Attribute	SOM1×1	SOM1×2	SOM1×3	SOM2×1	SOM2×2	SOM2×3	SOM3×1	SOM3×2	SOM3×3
NHR	0.03119	0.01588	0.00847	0.10806	0.02781	0.01514	0.40353	0.08253	0.03142
MDVP: Jitter: PPQ5	0.00314	0.00196	0.00141	0.00518	0.00276	0.00252	0.02569	0.00818	0.00437
DFA	0.66706	0.59746	0.59879	0.65859	0.61549	0.71544	0.62722	0.71986	0.73474
HNR	19.01099	23.71608	27.02710	13.93729	20.86188	22.78826	6.23466	16.39656	19.31760
Jitter: DDP	0.00867	0.00541	0.00391	0.01404	0.00766	0.00692	0.05760	0.02581	0.01210
RPDE	0.57058	0.52033	0.41584	0.66982	0.58463	0.52857	0.71176	0.65195	0.58484
MDVP: Jitter: RAP	0.00289	0.00180	0.00130	0.00468	0.00255	0.00231	0.01920	0.00860	0.00403
MDVP: Shimmer	0.05316	0.02264	0.01515	0.09985	0.03275	0.02199	0.16310	0.05656	0.03526
MDVP: Jitter (%)	0.00593	0.00395	0.00278	0.00941	0.00550	0.00492	0.03655	0.01600	0.00837
MDVP: Jitter (Abs)	0.00004	0.00003	0.00002	0.00007	0.00004	0.00004	0.00019	0.00013	0.00007
Shimmer:APQ5	0.03230	0.01300	0.00856	0.06125	0.01923	0.01271	0.10517	0.03199	0.02065
MDVP: Shimmer (dB)	0.48033	0.20956	0.13987	0.90445	0.30155	0.19930	1.44039	0.53211	0.32392
Shimmer:APQ11	0.04209	0.01880	0.01206	0.08172	0.02676	0.01866	0.11087	0.04382	0.03023
Shimmer:APQ3	0.02844	0.01123	0.00748	0.05062	0.01667	0.01070	0.07838	0.02874	0.01741
PPE	0.24002	0.16772	0.12162	0.31771	0.22379	0.20276	0.47903	0.41251	0.30025
Shimmer:DDA	0.08532	0.03370	0.02243	0.15185	0.05001	0.03210	0.23513	0.08622	0.05222

**Table 4:** Adjusted R^2^ and RMSE results for Motor-UPDRS 0.9489 and 0.5144; 0.9516, 0.5105.

Performance Index		SOM + Laplacian Score + Gaussian Process Regression (Exponential Kernel)		SOM + Laplacian Score + Gaussian Process Regression (Squared-Exponential Kernel)		SOM + Laplacian Score + Gaussian Process Regression (Rational Quadratic Kernel)		SOM + Laplacian Score + Gaussian Process Regression (Matérn 5/2 Kernel)	
		Train	Test	Train	Test	Train	Test	Train	Test
RMSE	Max	0.5184	0.5413	0.5734	0.5788	0.6056	0.6160	0.6439	0.6569
	Min	0.5104	0.5231	0.5436	0.5628	0.5769	0.5930	0.6121	0.6324
	Mean	0.5144	0.5322	0.5585	0.5708	0.5912	0.6045	0.6280	0.6447
$R_{adjusted\;}^{2}$	Max	0.9532	0.9320	0.9221	0.9180	0.9175	0.9135	0.9074	0.8967
	Min	0.9445	0.9234	0.9113	0.8932	0.8795	0.8554	0.8343	0.8103
	Mean	0.9489	0.9277	0.9167	0.9056	0.8985	0.8844	0.8708	0.8535

**Table 5:** Adjusted R^2^ and RMSE results for Total-UPDRS.

Performance Index		SOM + Laplacian Score + Gaussian Process Regression (Exponential Kernel)		SOM + Laplacian Score + Gaussian Process Regression (Squared-Exponential Kernel)		SOM + Laplacian Score + Gaussian Process Regression (Rational Quadratic Kernel)		SOM + Laplacian Score + Gaussian Process Regression (Matérn 5/2 Kernel)	
		Train	Test	Train	Test	Train	Test	Train	Test
RMSE	Max	0.5161	0.5379	0.5675	0.5742	0.6051	0.6139	0.6388	0.6516
	Min	0.5050	0.5216	0.5382	0.5564	0.5731	0.5872	0.6110	0.6307
	Mean	0.5105	0.5297	0.5529	0.5653	0.5891	0.6006	0.6249	0.6412
$R_{adjusted\;}^{2}$	Max	0.9565	0.9338	0.9287	0.9259	0.9246	0.9204	0.9157	0.8977
	Min	0.9468	0.9318	0.9190	0.8967	0.8863	0.8592	0.8406	0.8120
	Mean	0.9516	0.9328	0.9239	0.9113	0.9055	0.8898	0.8781	0.8548

## Data Availability

The data are available in the UCI machine learning archive, which was published online in 2009.

## Appendix A

**Table A1:** The results for Laplacian score.

Cluster 1	Cluster 2	Cluster 3	Cluster 4	Cluster 5	Cluster 6	Cluster 7	Cluster 8	Cluster 9
HNR	HNR	HNR	HNR	HNR	HNR	HNR	HNR	HNR
RPDE	RPDE	RPDE	NHR	RPDE	RPDE	NHR	MDVP:Shimmer (dB)	RPDE
MDVP:Shimmer (dB)	MDVP:Shimmer (dB)	DFA	MDVP:Shimmer (dB)	MDVP:Shimmer (dB)	MDVP:Shimmer (dB)	MDVP:Shimmer (dB)	MDVP:Shimmer	MDVP:Shimmer (dB)
NHR	NHR	Shimmer:APQ5	MDVP:Shimmer	PPE	MDVP:Shimmer	Shimmer:APQ5	Shimmer:APQ5	MDVP:Shimmer
MDVP:Shimmer	MDVP:Shimmer	MDVP:Shimmer (dB)	Shimmer:APQ5	MDVP:Shimmer	Shimmer:APQ5	MDVP:Shimmer	Shimmer:DDA	Shimmer:APQ5
PPE	PPE	MDVP:Shimmer	Shimmer:DDA	Shimmer:DDA	Shimmer:DDA	MDVP:Jitter:PPQ5	Shimmer:APQ3	Shimmer:DDA
DFA	DFA	Shimmer:APQ3	Shimmer:APQ3	Shimmer:APQ3	Shimmer:APQ3	Shimmer:APQ3	Shimmer:APQ11	Shimmer:APQ3
Shimmer:APQ5	Shimmer:APQ5	Shimmer:DDA	Shimmer:APQ11	Shimmer:APQ5	Shimmer:APQ11	Shimmer:DDA	NHR	PPE
Shimmer:APQ3	Shimmer:APQ3	MDVP:Jitter:PPQ5	RPDE	DFA	PPE	MDVP:Jitter (Abs)	PPE	Shimmer:APQ11
Shimmer:DDA	Shimmer:DDA	Shimmer:APQ11	DFA	Shimmer:APQ11	DFA	Shimmer:APQ11	DFA	DFA
Shimmer:APQ11	Shimmer:APQ11	PPE	PPE	MDVP:Jitter:PPQ5	MDVP:Jitter (Abs)	MDVP:Jitter (%)	RPDE	NHR
MDVP:Jitter (Abs)	MDVP:Jitter (Abs)	Jitter:DDP	MDVP:Jitter:PPQ5	NHR	NHR	Jitter:DDP	MDVP:Jitter:PPQ5	MDVP:Jitter (Abs)
MDVP:Jitter:PPQ5	MDVP:Jitter:PPQ5	MDVP:Jitter:RAP	MDVP:Jitter (%)	MDVP:Jitter (Abs)	MDVP:Jitter:PPQ5	MDVP:Jitter:RAP	MDVP:Jitter (%)	MDVP:Jitter (%)
MDVP:Jitter (%)	MDVP:Jitter (%)	MDVP:Jitter (%)	MDVP:Jitter:RAP	MDVP:Jitter (%)	MDVP:Jitter (%)	DFA	MDVP:Jitter (Abs)	MDVP:Jitter:PPQ5
MDVP:Jitter:RAP	MDVP:Jitter:RAP	MDVP:Jitter (Abs)	Jitter:DDP	Jitter:DDP	Jitter:DDP	PPE	MDVP:Jitter:RAP	Jitter:DDP
Jitter:DDP	Jitter:DDP	NHR	MDVP:Jitter (Abs)	MDVP:Jitter:RAP	MDVP:Jitter:RAP	RPDE	Jitter:DDP	MDVP:Jitter:RAP
